# Prenatal detection of distal 18p deletion by chromosomal microarray analysis: Three case reports and literature review

**DOI:** 10.1097/MD.0000000000039046

**Published:** 2024-07-26

**Authors:** Tangfei Xu, Fagui Yue, Jing He, Hongguo Zhang, Ruizhi Liu

**Affiliations:** a Center for Reproductive Medicine, Center for Prenatal Diagnosis, First Hospital, Jilin University, Changchun, China; b Jilin Engineering Research Center for Reproductive Medicine and Genetics, Jilin University, Changchun, China.

**Keywords:** 18p deletion, chromosomal microarray analysis, noninvasive prenatal testing, prenatal diagnosis

## Abstract

**Background:**

Chromosome 18p deletion syndrome is caused by total or partial deletion of the short arm of chromosome 18 and associated with cognitive impairment, growth retardation and mild facial dysmorphism. However, most studies on the genotype-phenotype correlations in the 18p region are diagnosed postnatally. Prenatal reports involving 18p deletions are limited.

**Methods:**

Three pregnant women opted for invasive prenatal testing due to noninvasive prenatal testing indicating high risk for chromosome 18 abnormalities. Karyotypic analysis and chromosomal microarray analysis (CMA) were performed simultaneously. The pregnancy outcomes for all cases were followed up. Meanwhile, we also made a literature review on prenatal phenotypes of 18p deletions.

**Results:**

G-banding analysis showed that 2 fetuses presented abnormal karyotypes: 45,XN,der(18)t(18;21)(p11; q11),-21 (case 2) and 46,XN,18p- (case 3). The karyotype of case 1 was normal. Meanwhile, CMA detected 4.37 Mb (case 1), 7.26 Mb (case 2) and 14.97 Mb (case 3) deletions in chromosome 18p region. All 3 pregnancies were terminated finally according to genetic counseling based upon abnormal CMA results.

**Conclusion:**

Prenatal diagnosis of 18p deletion syndrome is full of challenges due to the phenotypic diversity, incomplete penetrance and lack of prenatal phenotypes. Increased nuchal translucency and holoprosencephaly are common prenatal phenotypes of distal 18p deletion. For fetuses carrying 18p deletions with atypical sonographic phenotypes, noninvasive prenatal testing could be adopted as an effective approach.

## 1. Introduction

Chromosome 18p deletion syndrome, first described by de Grouchy et al, is a rare chromosomal disorder caused by total or partial deletion of the short arm of chromosome 18.^[1]^ The incidence of chromosome 18p deletion syndrome is estimated to be 1 in 50,000 newborn infants.^[2]^ In patients with del(18p) syndrome, about two-thirds of the cases have a *de novo* pure terminal deletion, while one-third of the cases are caused by a *de novo* unbalanced translocation, malsegregation of a parental translocation or inversion, or a ring chromosome or isochromosome 18q.^[3]^ The clinical phenotypes of chromosome 18p deletion syndrome is wide due to the diverse deleted fragments and the breakpoints. Patients with 18p deletion could present distinct phenotypes, including cognitive impairment, growth retardation, and mild facial dysmorphisms.^[4]^

For decades, karyotyping has been regarded as the gold standard for detecting chromosome abnormalities in prenatal diagnosis. However, this technique can hardly identify chromosomal aberrations smaller than 5 Mb due to its low resolution. With the development of molecular genetic testing technology, chromosomal microarray analysis (CMA) has gradually become the main diagnostic test method for genetic evaluation due to higher resolution and rapid detection ability.^[5]^ In recent years, some reports involving 18p deletion in a molecular level have been gradually reported worldwide.

Till now, most studies on the genotype–phenotype correlations in the 18p region were diagnosed postnatally. Prenatal reports involving 18p deletions were limited. Given the rare prenatal reports and diverse clinical manifestations of this syndrome, prenatal diagnosis for this chromosome disorder is still crucial and challenging. Herein, we present 3 cases of prenatal diagnostic 18p deletions with abnormal NIPT results and provide a systematic summary of prenatal phenotypes for such genomic disorders.

## 2. Methods

Our study protocol was approved by the Ethics Committee of the First Hospital of Jilin University (2021-706), and the written informed consents were obtained from the couples for publication of this case report and accompanying images.

### 2.1. Cytogenetic analysis

Chromosomal karyotypic analysis with a resolution of 300 to 400 bands was performed on G-band metaphases prepared from cultured aminotic fluid cells and peripheral blood cells according to standard protocols in our laboratory. Twenty metaphases were analyzed for all samples. The International System for Human Cytogenetic Nomenclature (ISCN 2016) nomenclature was used to describe all karyotypes.^[6]^

### 2.2. CMA

Genomic DNA was extracted from 10mL uncultured amino fluid cells using the QIAamp DNA Mini Kit (QIAGEN, Hilden, Germany) according to the manufacturer’s protocol and our previous study.^[7]^ The CMA was performed using the CytoScan 750K array (Affymetrix, Santa Clara, CA, USA), which included genomic DNA extraction, digestion and ligation, PCR amplification, PCR product purification, quantification and fragmentation, labeling, array hybridization, washing and scanning. Thresholds for genomewide screening were set at ≥ 200kb for gains, ≥100kb for losses. The detected copy number variations were comprehensively estimated by comparing them with published literature and the public databases: (1) Database of Genomic Variants (http://dgv.tcag.ca/dgv/app/home), (2) DECIPHR (https://www.deciphergenomics.org/), (3) ISCA (https://www.iscaconsortium.org/), (4) ECARUCA (http://www.ecaruca.net) and (5) online Mendelian inheritance in man (OMIM) (http://www.ncbi.nlm.nih.gov/omim). Genomic positions refer to the Human Genome Assembly Dec. 2013 (GRCh38/hg38).

## 3. Case presentation

### 3.1. Case 1

**Figure 1.**
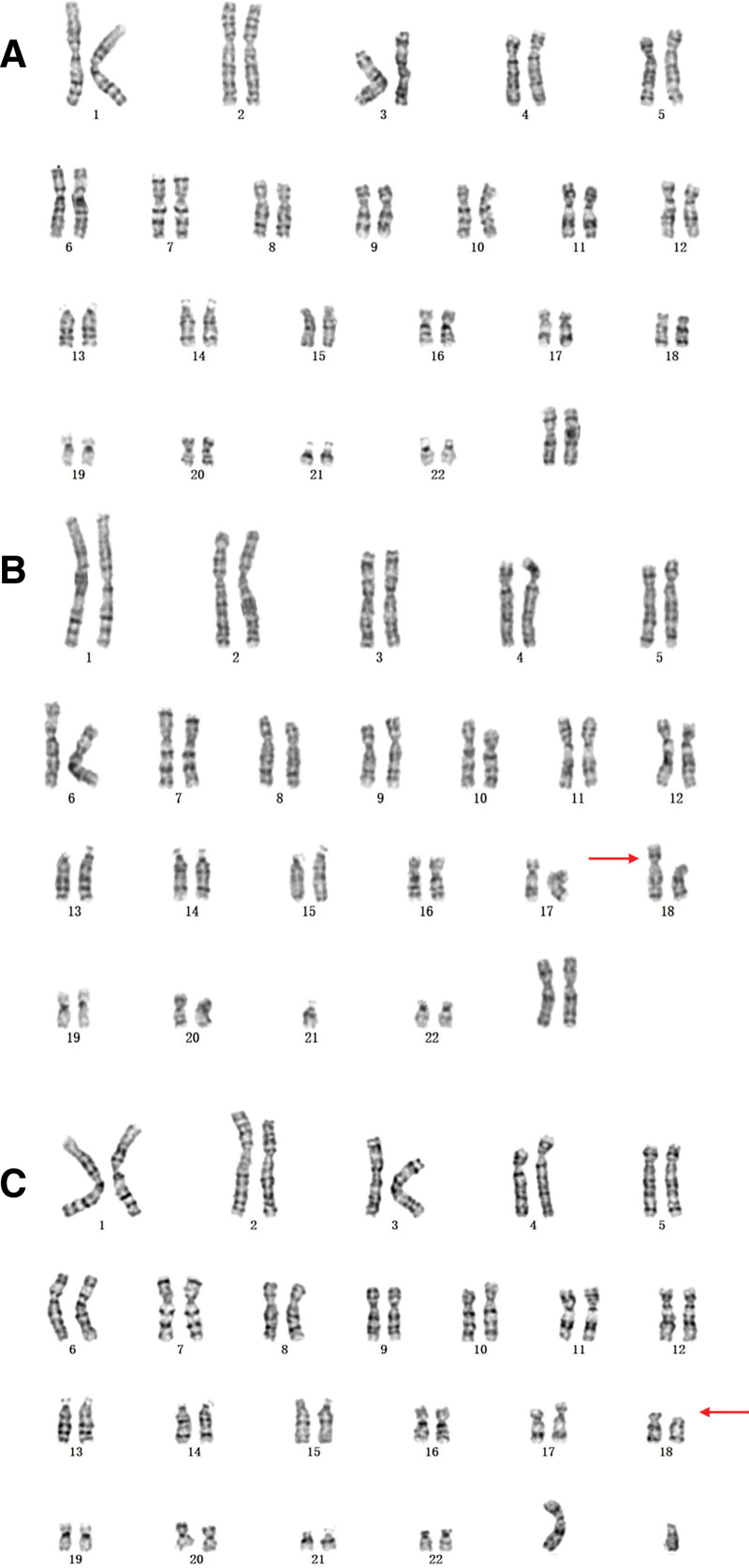
The results of karyotype analysis in our case. (A) The results of karyotype analysis in case 1: 46,XN. (B) The results of karyotype analysis in case 2: 45,XN,der(18)t(18;21)(p11;q11),-21. (C) The results of karyotype analysis in case 3: 46,XN,18p-.

A 33-year-old, gravida 2, para 1, pregnant woman underwent noninvasive prenatal testing (NIPT) at 21 weeks of gestation, which indicated high risk of chromosome 18 in the fetus. Subsequently, the woman underwent amniocentesis for cytogenetic analysis and CMA detection. G-banding analysis showed that the karyotype of the fetus was 46,XN (Fig. 1A). Meanwhile, CMA revealed a 4.37 Mb deletion in the region of 18p11.32p11.31. To identify the origin of this deletion, CMA detection for the couple was recommended. Unfortunately, the couple declined further genetic testing and chose to terminate the pregnancy at 23 weeks’ gestation.

### 3.2. Case 2

A 36-year-old, gravida 1, para 0, pregnant woman underwent NIPT at 19 weeks of gestation, which high risk of chromosome 18 in the fetus. Subsequently, the woman underwent amniocentesis for cytogenetic analysis and CMA detection. G-banding analysis showed that the karyotype of the fetus was 45,XN,der(18)t(18;21)(p11;q11),-21 (Fig. 1B). Meanwhile, CMA revealed a 7.26 Mb deletion in the region of 18p11.32p11.23. Subsequently, the couple accepted karyotype analysis to determine the origin of the variant. The karyotype analysis showed that their karyotypes were both normal, which indicated that the deletion in the fetus was *de novo*. Finally, the couple chose to terminate the pregnancy at 21 weeks’ gestation according to genetic counseling.

### 3.3. Case 3

A 26-year-old, gravida 1, para 0, pregnant woman underwent NIPT at 18 weeks of gestation, which indicated high risk of chromosome 18 in the fetus. Subsequently, the woman underwent amniocentesis for cytogenetic analysis and CMA detection. G-banding analysis showed that the karyotype of the fetus was 46,XN,18p- (Fig. 1C), and CMA revealed a 14.97 Mb deletion in the region of 18p11.32p11.21. Meanwhile, the couple opted for CMA detection for further verification. The results of CMA for the couple were normal, indicating that the deletion *de novo*. Finally, the couple chose to terminate the pregnancy at 20 weeks’ gestation according to genetic counseling based upon abnormal CMA results.

## 4. Discusstion

In our study, we reported 3 prenatal cases carrying 18p deletions using CMA and karyotypic analysis. Case 1 carried a 4.37 Mb deletion in the 18p11.32p11.31 region. Case 2 had a 7.26 Mb deletion in the 18p11.32p11.23 region. Case 3 had a 14.97 Mb deletion in the 18p11.32p11.21 region. No abnormal ultrasound findings were observed in these cases during the pregnancy periods.

18p deletion syndrome (OMIM: #146390), an uncommon chromosomal submicroscopic imbalance, is characterized by mental retardation, growth retardation, craniofacial dysmorphism (round face, short protruding philtrum, palpebral ptosis, dysplastic ears, wide mouth and dental anomalies) and abnormalities of the limbs, genitalia, brain, and heart. Till now, more than 300 cases have been reported worldwide, but most of them are postnatal cases, and prenatal reports are particularly rare. 18p deletions are usually detected through chorionic villus sampling or amniocentesis in prenatal settings. Moreover, it is notable that some clinic features of 18p deletions can hardly be detected in prenatal ultrasound examinations.

**Figure 2.**
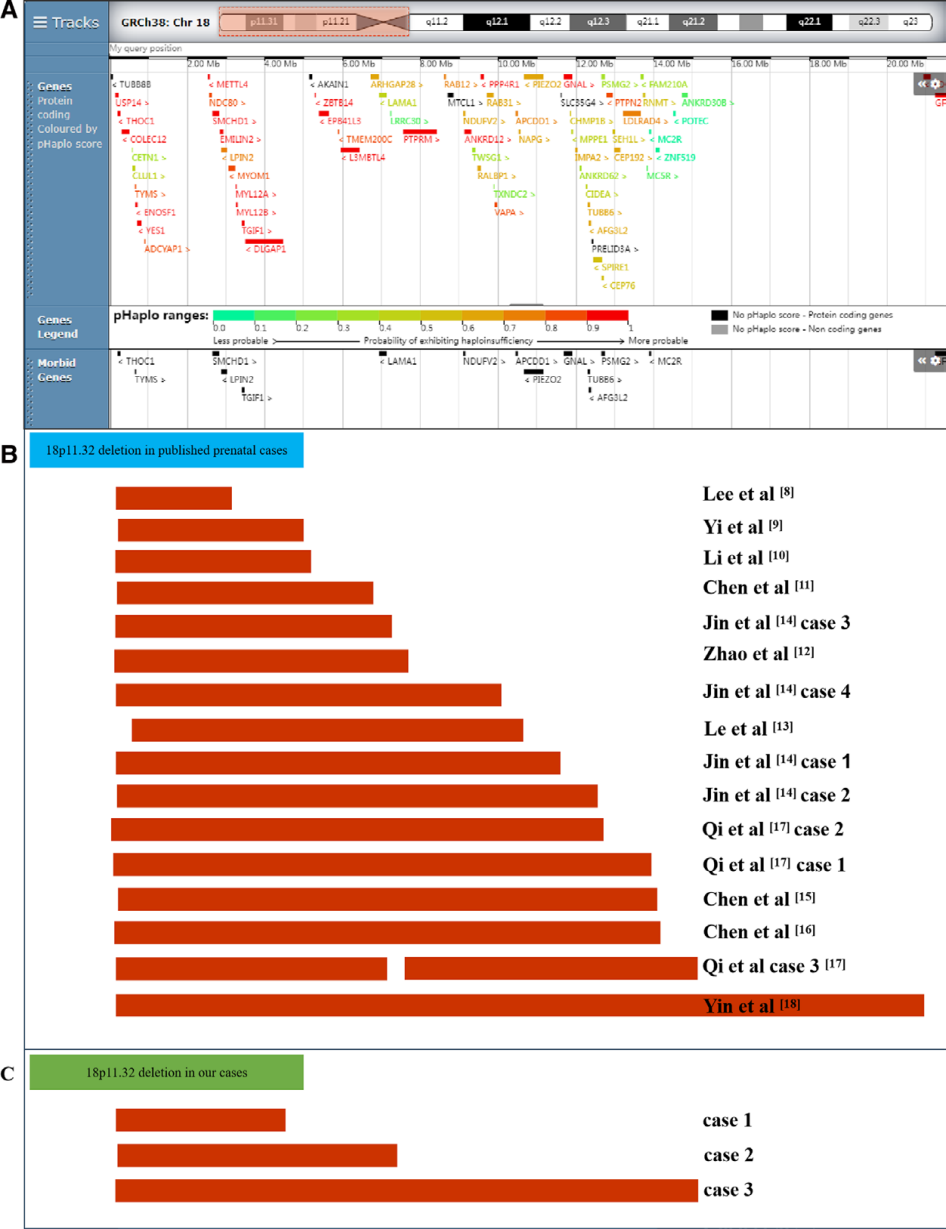
Scale representation of the deleted region in the short arm of chromosome 18p11.32 (https://www.deciphergenomics.org/): (A) Genes involved in the 18p11.32 region; (B) previously described 18p11.32 deletions in the prenatal period; and (C) deleted fragments in our cases. Genomic parameters are from GRCh38/hg38.

In this study, we report 3 prenatal cases carrying distal 18p deletions, spanning from 4.37 Mb to 14.97 Mb. In order to establish a better understanding on the antenatal phenotypes, we summarized the clinical manifestations of prenatal cases sharing similar 18p11.32 deletion with our cases according to literature review (Table 1, Fig. 2).^[8–18]^ All 18p11.32 microdeletions were varied in size, from 3 Mb to 18 Mb. Among these deletions, 4/19 were parentally inherited, 12/19 cases were *de novo*, and 3/19 cases were not available. Among them, 12/19 cases presented ultrasound abnormalities. Increased nuchal translucency (NT) was observed in 4/19 cases and holoprosencephaly (HPE) was discovered in 5/19 cases. Besides, hydronephrosis was observed in 2/19 cases and cardiac abnormality was discovered in 1/19 cases. It was worth noting that the remaining 7/19 cases without ultrasound abnormalities were detected using NIPT (6/19) and maternal serum screening (1/19). The termination of pregnancy was opted for all cases except case 6. Based upon the fact mentioned above, increased NT and HPE are common prenatal phenotypes of distal 18p deletion, and NIPT plays a critical role in detecting fetuses with 18p deletions presenting no abnormal ultrasound findings.

**Table 1:** The prenatal phenotypes of published literature and present cases with 18p deletion.

No.	Sex	Age	Gravida and para	Gestational age (wk)	Indications for prenatal diagnosis	Parental phenotypes	Deleted region	Deleted size (Mb)	Inheritance	Karyotype	CMA results (GRCh38)	Pregnancy outcome	References
1	F	37	G2P0	20+	Ultrasound findings inferred bilateral hydronephrosis and the presence of 2 choroid plexus cysts (CPCs) at 17 wk and 20 wk	Normal	18p11.32p11.31	3	pat	46,XX,rec(18)dup(18q)inv(18)(p11.2q21.2)pat	18p11.32p11.31 (136,227–3,100,355) × 1	TOP at 23 wk 3d	Lee et al^[8]^
2	M	29	G1P0	24+	Ultrasound findings inferred semilobar holoprosencephaly, median cleft lip and palate, arhinia, and tetralogy of fallot	Normal	18p11.32p11.31	4.5	*de novo*	46,XY	18p11.3211.31 (136,226–4,925,311) × 1	TOP	Yi et al^[9]^
3	F	28	G1P0	20+	NIPT indicated a high risk of chromosome 18 microdeletion; Ultrasound findings inferred FGR and ventricular septal defect and persistent truncus arteriosus	Normal	18p11.32p11.31	5	*de novo*	46,XX,18p+	18p11.32p11.31 (136,227–5,159,806) × 1	TOP	Li et al^[10]^
4	NA	26	G1P0	19+	NIPT indicated a high risk of chromosome 18; normal ultrasound examination	Normal	18p11.32p11.31	6.66	mat	46,XN,del(18)(p11.3)	18p11.32p11.31 (136,226–6,796,179) × 1	TOP at 33 wk	Chen et al^[11]^
5	F	20	G1P0	20+	NIPT indicated partial or complete deletion of the X chromosome	Normal	18p11.32p11.23	7.56	*de novo*	46,XX	18p11.32p11.23 (120,000–7,680,002) × 1	TOP	Zhao et al^[12]^
6	F	NA	G2P1	NA	Maternal serum screening inferred high risk of trisomy 21; increased NT (3.9 mm)	Normal	18p11.32p11.22	9.9	*de novo*	46,XX,18p-	18p11.32p11.22 (618,247–10,597,242) × 1	Born at term (birth weight: 3000 g)	Le et al^[13]^
7	NA	26	G1P0	NA	Abnormal childbearing history (child with HPE)	Mother: round face, thick neck, mild language development delay	18p11.32p11.21	11.46	mat	NA	18p11.32p11.21 (120,001–11,580,001) × 1	TOP at 24 wk	Jin et al^[14]^
8	F	40	G2P1	26+	Ultrasound findings inferred a severe hydronephrosis	Normal	18p11.32p11.21	12.39	NA	46,XX,del(18)(p11.21)	18p11.32p11.21 (146,484–12,532,805) × 1	TOP	
9	F	28	G2P1	22+	NIPT indicated a high risk of chromosome 18	Normal	18p11.32p11.23	7.1	NA	46,XX,del(18)(?p11.2)	18p11.32p11.23 (146,484–7,244,644) × 1	TOP	
10	F	31	G2P1	19+	Increased NT (3.5 mm)	Normal	18p11.32p11.22	9.9	*de novo*	46,XX,del(18)(p11.2)	18p11.32p11.22 (146,484–10,048,315) × 1	TOP	
11	F	32	G2P1	19+	Maternal serum screening inferred high risk of trisomy 18; ultrasound findings inferred alobar HPE	Normal	18p11.32p11.21	14	*de novo*	46,XX,i(18)(q10)	18p11.32p11.21 (138,992–14,071,858) × 1	TOP	Chen et al^[15]^
12	F	36	G2P1	19+	Advanced maternal age, ultrasound findings inferred craniofacial abnormalities, HPE and median facial cleft	Normal	18p11.32p11.21	14.06	*de novo*	46,XX,del(18)(p11.21)dn	18p11.32p11.21 (64,223–14,124,701) × 1	TOP	Chen et al^[16]^
13	M	32	G4P0	20+	Ultrasound findings inferred increased NT (5.1 mm), nuchal fold (NF = 11 mm), FGR and oligohydramnios	Normal	18p11.32p11.21	13.87	*de novo*	46,XY,del(18)(cen→qter)	18p11.32p11.21 (14,316–13,885,256) × 1	TOP at 34 wk	Qi et al^[17]^
14	F	32	G2P0	18+	Maternal serum screening inferred high risk for trisomy 21	Normal	18p11.32p11.21	12.68	*de novo*	46,XX,15p+,18p-	18p11.32p11.21 (14,316–12,691,472) × 1	TOP at 32 wk	
15	NA	31	G1P0	18+	Increased NT (4.9 mm) combined FGR	Normal	18p11.32p11.31 18p11.23p11.21	6.9 7.5	*de novo*	46,XN,del(18)(p11.2)	18p11.32p11.31 (136,227–7,080,665) × 1 18p11.23p11.21 (7,579,981–15,170,637) × 1	TOP at 23 wk	
16	F	24	G3P0	23+	Ultrasound findings inferred fused cerebral hemispheres, dilatation of the cerebral ventricles, a single palpebral fissure and proboscis	Normal	18p11.32q11.1	18	mat	46,XX,der(18)t(18;21)(p11;p11) mat	18p11.32q11.1 (136,226–20,941,324) × 1	TOP	Yin et al^[18]^
17	NA	33	G2P1	21+	NIPT indicated a high risk of chromosome 18; no abnormal ultrasound findings were observed	Normal	18p11.32p11.31	4.37	NA	46,XN	18p11.32p11.31 (136,227–4,504,956) × 1	TOP at 23 wk	Our case 1
18	NA	36	G1P0	19+	NIPT indicated a high risk of chromosome 18; no abnormal ultrasound findings were observed	Normal	18p11.32p11.23	7.26	*de novo*	45,XN,der(18)t(18;21)(p11;q11),-21	18p11.32p11.23 (136,227–7,396,531) × 1	TOP at 21 wk	Our case 2
19	NA	26	G1P0	18+	NIPT indicated a high risk of chromosome 18; no abnormal ultrasound findings were observed	Normal	18p11.32p11.21	14.97	*de novo*	46,XN,18p-	18p11.32p11.21 (136,227–15,106,306) × 1	TOP at 20 wk	Our case 3

d = days, F = female, FGR = fetal growth restriction, HPE = holoprosencephaly, M = male, mat = maternally inherited, NA = not available, NIPT = noninvasive prenatal testing, NT = nuchal translucency, pat = paternally inherited, TOP = termination of pregnancy, wk = weeks; Genomic parameters are from GRCh38/hg38.

According to the DECIPHER database, 5 morbid genes are located in the 18p11.32 region detected in our cases (Table 2), which are correlated with diverse diseases. As known, the haploinsufficiency for genes would result in genetic disorders. In order to predict the potential genes associated with poor prognostic phenotypes, we delineated their functions and implications in different processes.

**Table 2:** Overlapping genes in the 18p deletion region in our case.

Gene	OMIM	Description	Disease
*THOC1*	606930	THO complex subunit 1	Deafness, autosomal dominant 86
*TYMS*	188350	Thymidylate synthetase	Dyskeratosis congenita, digenic
*SMCHD1*	614982	Structural maintenance of chromosomes flexible hinge domain containing 1	Facioscapulohumeral Muscular Dystrophy 2 Bosma Arhinia Microphthalmia Syndrome
*LPIN2*	605519	Lipin 2	Majeed syndrome
*TGIF1*	602630	Transforming growth factor-beta-induced factor	Holoprosencephaly 4

*TGIF1* (OMIM:602630) encodes transforming growth factor-β-induced factor, which belongs to a family of evolutionarily conserved, atypical homeodomain proteins that act as transcriptional repressors and co-repressors in retinoid and transforming growth factor signaling pathway.^[19]^ *TGIF1* has been proposed as a candidate gene which is implicated in the etiology of HPE in patients with chromosome 18p deletions. Reduced *TGIF1* levels would enhance the binding of retinoid X receptor (RXR) to retinoid responsive promoters, resulting in overactivity of retinoic acid-regulated genes and simulating the effect of excess retinoic acid exposure.^[20–22]^ *SMCHD1* (OMIM: 614982), containing a structural maintenance of chromosomes (SMC) hinge domain, plays a role in epigenetic silencing.^[23–25]^ Heterozygous variants of *SMCHD1* have been associated with facioscapulohumeral muscular dystrophy (FSHD) and the rare craniofacial disorder Bosma arhinia microphthalmia syndrome.^[26–28]^ *TYMS* (OMIM: 188350) encodes thymidylate synthase, which is essential for DNA replication and repair.^[29]^ Polymorphisms in this gene are associated with an increased risk of conotruncal cardiac diseases and colorectal cancer.^[30–32]^ *THOC1* and *LPIN2* genes have been rarely studied in patients carrying 18p deletions, and their functions need to be further investigated.

As a promising technique, NIPT has been frequently applied to determine fetal sex, aneuploidies, microdeletions/microduplications in prenatal screening in recent years.^[33]^ In our study, NIPT indicated a high risk of chromosome 18 for all pregnancies, which further prompted the pregnant women to opt for prenatal diagnosis and ultimately led to the detection of 18p deletion.

In our study, all couples chose to terminate their pregnancies based on genetic counseling. For case 2 and case 3 couples, preimplantation genetic testing was an ideal option and prenatal diagnosis is necessary if they intended to conceive again. For case 1, CMA testing was still necessary to determine whether the 18p deletion was *de novo* or inherited from the parents.

## 5. Conclusion

We describe 3 prenatally diagnosed cases with 18p deletion by karyotype and CMA. Fetuses with 18p microdeletions could exhibit diverse ultrasound findings, ranging from normal to abnormal. Increased NT and HPE are common prenatal phenotypes of distal 18p deletion. In addition, NIPT could be adopted as an effective approach in detecting 18p deletion in prenatal setting, especially when no abnormal ultrasound findings were observed. Our study would provide a better prenatal management for the genetic detection and clinic counseling of 18p deletions.

## Author contributions

**Conceptualization:** Xu Tangfei, Rui-Zhi Liu.

**Writing – original draft:** Xu Tangfei.

**Data curation:** Yue Fagui.

**Formal analysis:** He Jing.

**Methodology:** Zhang Hongguo.

**Supervision:** Rui-Zhi Liu.
